# Safety, Efficacy, and Intermediate-Term Outcomes of Radiofrequency Catheter Ablation for Pediatric Arrhythmias

**DOI:** 10.7759/cureus.10488

**Published:** 2020-09-16

**Authors:** Shunmuga Sundaram Ponnusamy, Giridhar Muthu, Mahesh Kumar, Dasarath Bopanna, Vijesh Anand, Suryakumar Balasubramanian

**Affiliations:** 1 Cardiology, Velammal Medical College Hospital and Research Institute, Madurai, IND

**Keywords:** radio-frequency ablation, supraventricular tachycardia, pediatric arrhythmia, atrioventricular nodal re-entrant tachycardia

## Abstract

Background

Arrhythmias are one of the reasons for emergency hospitalization among the pediatric population. Radiofrequency ablation is a major advancement in the management of children with cardiac arrhythmias.

Objectives

Our study was designed to describe the outcomes of catheter ablation in the pediatric population for atrial and ventricular arrhythmias in our center.

Methods

All patients between two and 18 years of age undergoing radiofrequency ablation after failed medical management for arrhythmias in our institute were included. Age less than two years, no previous medical management, and complex congenital heart disease were excluded. Baseline and electrophysiological characteristics were recorded. The patients were followed up for a clinical or electrocardiographic recurrence of arrhythmia.

Results

Thirty-six patients were included (mean age 12.8 ± 3.9 years (range 2-18 years), male 56%, average weight 36.39 ± 11.02 kg). The mean follow-up duration was 27.7 ± 15.9 months (range 3-58). Five-point five percent (5.5%) had tachycardiomyopathy. The arrhythmias included (1) atrioventricular nodal re-entrant tachycardia (AVNRT, n=16, 44%), (2) atrioventricular reciprocating tachycardia (AVRT, n=14, 39%), (3) atrial tachycardia (AT, n=2, 5.5%), (4) ventricular premature complexes (VPCs, n=2, 5.5%), (5) atrial flutter (AFL, n=1, 3%), and (6) ventricular tachycardia (VT, n=1, 3%). A transeptal puncture was done in 10 patients (28%). 3D mapping was done in six patients. The mean radiofrequency (RF) pulses were 2.3 ± 1.3. The acute procedural success rate was 100%. The long-term success was 97.2%. One (2.7%) developed recurrence. No major complications were reported.

Conclusion

Catheter ablation in the pediatric population is a safe procedure and can be done with more feasibility and fewer complications when done in experienced hands.

## Introduction

Arrhythmias in the pediatric population are not uncommon. Around 5% of emergency admissions are attributed to cardiac arrhythmias [1]. As the incidence of congenital heart disease (CHD) is on the rise in the pediatric population, they are more prone to arrhythmias after surgical correction of the complex CHD. Supraventricular tachycardia (SVT) is the most common tachyarrhythmia that occurs, and it constitutes around 90% of the total arrhythmic burden [2]. Atrioventricular reciprocating tachycardia (AVRT) constitutes 85% of fetal arrhythmias and 82% of arrhythmias during infancy. Most of these arrhythmias resolve before two years though there may be late recurrence [3]. Atrioventricular nodal re-entrant tachycardia (AVNRT) constitutes 23% of arrhythmias in the one to five age group, 35% in the six to 10 age group, and 20% in those over 10 years of age. AVNRT rarely resolves spontaneously and will need radiofrequency ablation [4]. The incidence of atrial tachycardia (AT) is around 15% during childhood, and some may need ablation. Up to one-third of neonates have ventricular premature complexes (VPCs) and usually resolve by two weeks of age. If they persist beyond that period, further investigations are required.

Most of the supraventricular arrhythmias in children respond to medical management, and radio-frequency (RF) ablation can be reserved for refractory cases [5]. Safety and long-term success had been shown in various prospective and retrospective studies [6-8]. Recent guidelines recommend electrophysiological study and catheter ablation to be extended to the pediatric population as in adults [9-10]. Despite its procedural success and low rate of complications, catheter ablation for the pediatric population is performed less in number owing to its demand of high technical expertise and lack of experience. The aim of our study is to describe the baseline characteristics and outcomes in the pediatric population who underwent catheter ablation for atrial and ventricular arrhythmias in our center.

## Materials and methods

Study design

It is a single-center, retrospective observational study done among patients less than 18 years of age who underwent electrophysiological study and radiofrequency ablation from June 2015 to January 2020 at Velammal Medical College Hospital and Research Institute. The study was conducted after getting approval from the institutional ethical committee. Informed consent for the procedure was obtained from the parents.

Patient selection

All patients between two and 18 years of age undergoing radiofrequency ablation after failed medical management for atrial and ventricular arrhythmias were included. These include AVNRT, AVRT, AT, AFL, VPCs, and VT. The confirmation of arrhythmia was based on 12-lead electrocardiography (ECG). The exclusion criteria included age less than two years, no previous trial of medical management, and the presence of complex congenital heart disease. A detailed clinical history was obtained from the parents of all patients, including palpitation, syncope, heart failure, medication intake, and a family history of cardiac arrhythmias. Echocardiography was done for all patients, to rule out structural heart disease. The anti-arrhythmic medications were discontinued for five half-lives before the procedure.

Electrophysiological study and ablation

An electrophysiology study was done under general anesthesia after obtaining informed consent using intravenous ketamine 1 mg/kg and fentanyl 1 mcg/kg. Venous access was obtained using a modified Seldinger technique. A right internal jugular venous (IJV) puncture was obtained for placing a decapolar catheter into the coronary sinus. Two femoral venous access was obtained on the right side and one on the left side for placing the right ventricle (RV), the His bundle (HB), and the ablation catheter. In patients less than five years of age, IJV access was avoided, and the decapolar catheter was placed from the femoral vein. After obtaining venous access, intravenous heparin was given at a 100 IU/kg dose.

Twelve-lead ECG and intracardiac electrograms were continuously recorded using a St. Jude electrophysiology system. Baseline intervals, including sinus cycle length, AH, and HV intervals, were recorded. The standard institutional protocol was used for arrhythmia induction. Incremental pacing and programmed stimulation done from the right atrium and right ventricle were used to find out (1) the antegrade and retrograde refractory period of the atrioventricular node (AV node), (2) the effective refractory period of the atrium, (3) the effective refractory period of the ventricle, and (4) the induction of arrhythmia. If arrhythmia could not be induced then the protocols were repeated after giving isoprenaline infusion at the rate of 1-3 µg/kg. For patients undergoing ablation for atrial tachycardia, atrial flutter, and ventricular arrhythmias, three-dimensional electroanatomic mapping was used (EnSite Velocity, St Jude Medical, Saint Paul, Minnesota).

Once the narrow QRS tachycardia (defined as a QRS duration of less than 120 ms and a cycle length of less than 600 ms) was induced, the arrhythmia was classified as AVNRT, AVRT, atrial tachycardia, or atrial flutter based on the electrophysiological characteristics. Tachycardia cycle length, coronary sinus electrodes activation pattern, ventricular entrainment protocol, His refractory VPCs, and mode of termination of tachycardia were used to classify the tachycardia.

AVNRT was diagnosed when programmed atrial stimulation showed an AH jump of >50 ms, an echo beat, or sustained narrow QRS tachycardia. The tachycardia was differentiated from atrial tachycardia and AVRT by the ventricular entrainment protocol (VAAV response, post pacing interval (PPI) minus tachycardia cycle length (TCL) >115 ms, stimulus to atrial (SA) interval minus ventricle to atrial (VA) interval >85 ms). Ablation was done during sinus rhythm, targeting the posterior part of Koch's triangle just outside the coronary sinus ostium. RF pulses were given if the ablation catheter showed A:V signals at a 1:10 ratio with a fractionated atrial electrogram (slow pathway potential). Ablation was considered successful if accelerated junctional rhythm with 1:1 VA conduction occurred continuously for at least 20 seconds and failure to induce clinical arrhythmia after RF pulses with or without isoprenaline infusion.

AVRT was diagnosed when ventricular entrainment showed VAAV response, PPI-TCL <115 ms, SA-VA <85 ms, and VA fusion during tachycardia depending upon the site of the accessory pathway. Ablation was done during tachycardia in patients with a concealed bypass tract (with retrograde conduction only) and during sinus rhythm in patients with manifest pre-excitation. For left-sided pathways, the left atrium was entered by septal puncture using a Brockenbrough needle. Ablation was considered successful if VA or AV dissociation occur during ablation with the disappearance of manifest pre-excitation and failure to induce clinical arrhythmia after RF pulses with or without isoprenaline infusion.

For atrial tachycardia, atrial flutter, and ventricular tachycardia three-dimensional electroanatomical mapping (EnSite Velocity) was used. Precise creation of chamber geometry followed by ablation guided by activation and entrainment mapping was done.

Short-term and long-term outcomes

The acute success of the procedure is defined as the absence of inducible arrhythmias for 30 minutes after the application of the last ablation with or without isoprenaline infusion. Patients were discharged 24 hours after the procedure. Venous access sites were carefully evaluated before discharge. A six-monthly follow-up was done to look for the clinical recurrence of palpitation, documented tachycardia, or an asymptomatic recurrence of pre-excitation. Long-term success is defined as the absence of recurrence in patients with acute procedural success, until the end of follow-up.

Statistical evaluation

Continuous variables are reported as mean±standard deviation (SD) and compared with the two-tailed Student's t-tests, and categorical variables were compared using the chi-square test. All statistical tests were two-tailed; P<0.05 was considered to indicate statistical significance.

## Results

Baseline, procedural characteristics, and indications

Radiofrequency ablation for drug-refractory arrhythmias was done in 36 patients between June 2015 and January 2020. The baseline characteristics of the study population are given in Table 1. The mean age of the study population was 12.86 ± 3.95 years (range 2-18 yrs). Twenty patients were male (56%). The mean weight of the study population was 36.3 ± 11.02 kg. Patients were followed up for a duration of 27.7 ± 16.9 months (range 3-58 months). The predominant presenting complaint was palpitation (92%). Two patients (5.5%) had left ventricular dysfunction related to tachyarrhythmia (incessant focal atrial tachycardia and atrial flutter). None of our study patients had congenital heart disease. Six cases were done using the 3D EnSite Velocity system (two atrial tachycardias, two VPCs, one VT, and one atrial flutter).

**Table 1:** Baseline characteristics of the study population

Total number of patients	36
Male	20 (56%)
Female	16 (44%)
Age	12.86±3.95 years (2-18 years)
Weight	36.39±11.02 kg
Follow-up duration	27.75±15.98 months (3-58 months)
Structural heart disease	Nil
Palpitation	33 patients (92%)
Syncope	Nil
Left ventricular dysfunction	2 patients (5.5%)

The arrhythmias in the study population (Table 2) included (1) atrioventricular nodal re-entrant tachycardia (AVNRT) (n=16, 44%), (2) atrioventricular reciprocating tachycardia (AVRT) (n=14, 39%), (3) atrial tachycardia (AT) (n=2, 5.5%), (4) ventricular premature complexes (VPCs) (n=2, 5.5%), (5) atrial flutter (AFl) (n=1, 3%), and (6) ventricular tachycardia (VT) (n=1, 3%). Manifest pre-excitation was noted in nine patients.

**Table 2:** Arrhythmia profile in the study population VPCs: ventricular premature complexes

Total number of patients	36
Atrioventricular nodal re-entrant tachycardia	16 (44%)
Atrioventricular reciprocating tachycardia	14 (39%)
Left lateral	10
Right posteroseptal	2
Right mid-septal	1
Left posteroseptal	1
Atrial tachycardia	2 (5.5%)
Atrial flutter	1 (3%)
VPCs	2 (5.5%)
Ventricular tachycardia	1 (3%)

Electrophysiology study and radiofrequency ablation data

**Figure 1:**
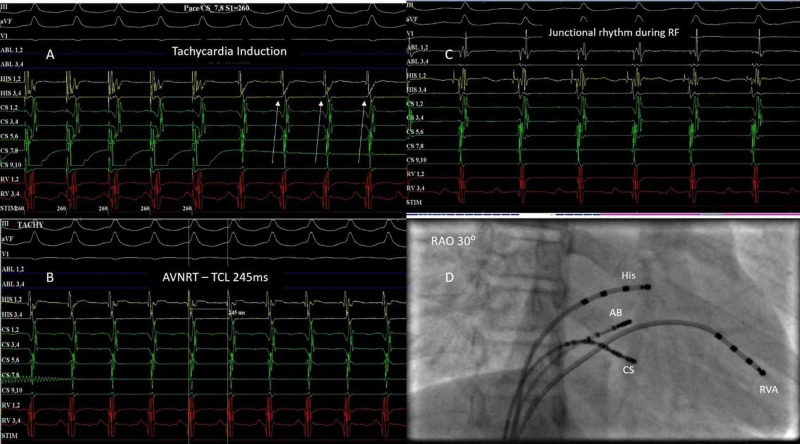
AVNRT – 12-year-old child with recurrent AVNRT Tachycardia induction with AH jump (A) by programmed atrial stimulation from coronary sinus (CS)-ostium with a cycle length of 245 ms (B). The white arrow indicates the activation pattern from proximal to distal CS. Accelerated junctional rhythm (C) noted during radiofrequency (RF) ablation (settings power 50W, temperature 50⁰C). Right anterior oblique fluoroscopic view (D) showing the position of the ablation catheter with reference to his bundle. AVNRT: atrioventricular nodal re-entrant tachycardia; TCL: tachycardia cycle length; AB: ablation catheter; CS: coronary sinus; RVA: right ventricle apex; His: His bundle catheter; RF: radiofrequency ablation

The electrophysiological characteristics of the study population are given in Table 3. In all the patients, the procedure was done under conscious sedation. The mean AH interval was 86.1 ± 7.9 ms. The mean HV interval was 38.1 ± 8.1 ms. The mean tachycardia cycle length (TCL) was 297.8± 30.4 ms. Sixteen patients underwent ablation for AVNRT (Figure 1). A medium curve ablation catheter was used to target the posterior half of Koch's triangle (Figure 1D). A transient accelerated junctional rhythm could be obtained in all patients (Figure 1C). To avoid injury to the AV node, the ablation pulses were interrupted immediately if a fast junctional rhythm at a cycle length of less than 350 ms occurs or if there was a drop in VA conduction. One of the patients was a two-year-old child with drug-refractory AVNRT requiring multiple hospitalizations. Single ablation given at the posterior part of Koch's triangle resulted in a good junctional rhythm, after which tachycardia was not inducible. None of our patients developed AV nodal injury during ablation.

**Table 3:** Electrophysiological and follow-up data

Electrophysiological characteristics	
Manifest pre-excitation	9 patients(25%)
Tachycardia cycle length	297.8±30.4 ms
3D EnSite mapping	6 patients(17%)
Septal puncture	10 patients(28%)
Number of ablations given	2.3±1.3
Acute success rate	100%(36/36)
Complications	
Thromboembolism	Nil
Perforation	Nil
Pneumothorax	Nil
Death	Nil
Recurrence	1(2.7%)

Among 14 patients with AVRT, 10 had the left lateral pathway, two had the right posteroseptal pathway, one had the right mid septal pathway, and one had the left posteroseptal pathway. Transeptal puncture was done for all left-sided accessory pathways (n=10). The atrial septum could be easily punctured with a Brockenbrough needle even in patients with age less than seven years (n=2 patients). No complications related to transseptal puncture were noted. Ablation was done during sinus rhythm in patients who had manifest pre-excitation.

**Figure 2:**
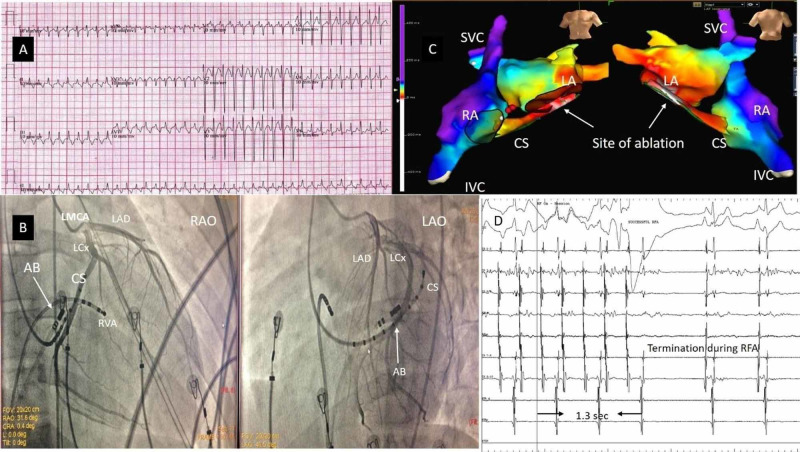
Atrial tachycardia A 17-year-old male patient with incessant atrial tachycardia (A) with left ventricular dysfunction. Coronary angiography was done (B) to confirm the location of the ablation catheter in relation to the coronary artery. LMCA: left main coronary artery; LAD: left anterior descending artery; LCx: left circumflex artery; CS: coronary sinus; AB: ablation catheter; RVA: right ventricular apex; LAO: left anterior oblique; RAO: right anterior oblique. Activation map (C) using EnSite showed the earliest activation at the mid-coronary sinus where ablation resulted in the termination of tachycardia (D) within 1.3 seconds. SVC: superior vena cava; IVC: inferior vena cava; LA: left atrium; RA: right atrium; CS: coronary sinus; RFA: radiofrequency ablation

**Figure 3:**
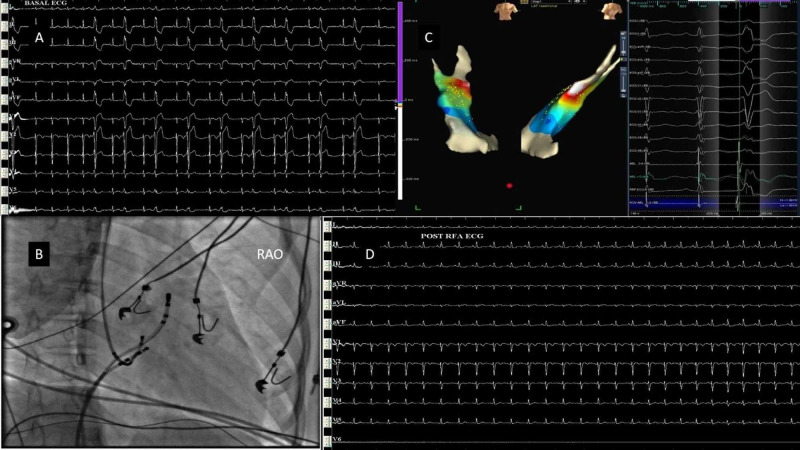
RVOT – VPCs A nine-year-old girl with RVOT-VPCs in bigeminal rhythm (A). EnSite (C) was used to ablate the VPC at the anterior septum (B). Post-procedure ECG showing normal sinus rhythm without VPCs. RVOT: right ventricular outflow tract; VPCs: ventricular premature complexes; RAO: right anterior oblique; RFA: radiofrequency ablation; ECG: electrocardiogram

**Figure 4:**
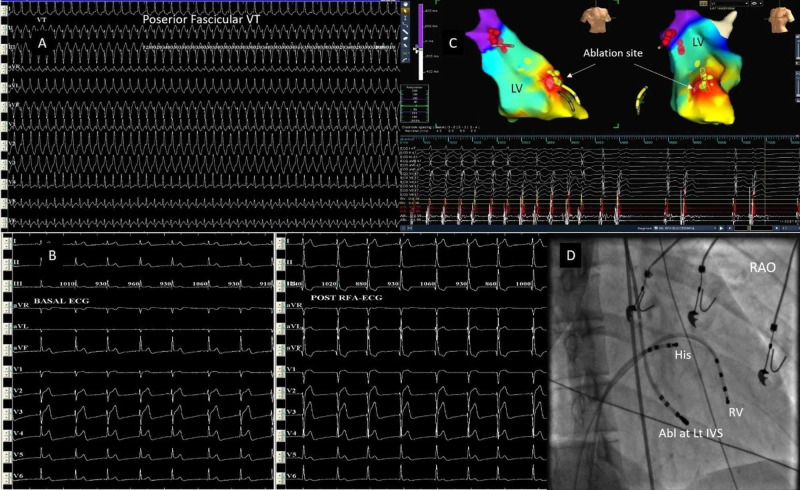
Posterior fascicular ventricular tachycardia An 18-year-old male patient with posterior fascicular ventricular tachycardia (A). 3D geometry was created and activation mapping confirmed the earliest site at the left distal IVS (C and D). Post-procedure ECG showed a left posterior fascicular block (q in inferior leads and rS in lead I and aVL) pattern. LV: left ventricle; RFA: radiofrequency ablation; RAO: right anterior oblique; HIS: the His bundle catheter; RV: right ventricle; ABL: ablation; Lt IVS: left interventricular septum

3D electroanatomic mapping using the EnSite Velocity system was used in six patients. 3D geometry was created during tachycardia. Ablation was guided by the activation and entrainment map. Two patients had atrial tachycardia - one from the coronary sinus (CS) muscle sleeve and the other arising from the coronary sinus ostium. The CS muscle sleeve tachycardia patient presented with incessant AT with left ventricular (LV) dysfunction (Figures 2A-2B). Ablation was done using an irrigated tip ablation catheter inside the mid coronary sinus (Figure 2C). One patient had a typical right atrial flutter with a fast ventricular rate and LV dysfunction. An isthmus ablation drawn using an irrigated tip ablation catheter resulted in the termination of tachycardia. Two patients had right ventricular outflow tract (RVOT)-VPCs occurring in bigeminal rhythm with ill-sustained VT (Figure 3). One of these patients had VPC from the pulmonary cusp, which was successfully ablated from below the cusp. One patient had posterior fascicular ventricular tachycardia, which was successfully ablated at the distal interventricular septum (IVS) close to its exit point using the irrigated tip ablation catheter (Figure 4).

The mean number of RF pulses needed for successful ablation was 2.3 ± 1.3 (excluding atrial flutter patients where an isthmus line was drawn from the tricuspid annulus to the inferior vena cava at the 6 o'clock position in the left anterior oblique (LAO) fluoroscopic view).

The acute procedural success rate of catheter ablation for 36 patients in our study is 100%. None of our patients had inducible tachycardia, AH jump/echo beat, or persistence of pre-excitation at the end of the study. No AV node conduction defects occurred on short- and long-term follow-up.

The long-term success rate in our series is 97.2%. One patient with Wolff-Parkinson-White (WPW) syndrome with the right posteroseptal pathway had an asymptomatic recurrence of the pathway after two months. Both tachycardiomyopathy patients improved on follow-up, and the ejection fraction recovered back to normal. The reported recurrence rate of arrhythmia after RF ablation in our series of patients is 2.7% (one patient). No thromboembolism, cardiac perforation, pneumothorax, or death occurred.

## Discussion

Arrhythmias are one of the less frequently encountered problems among the pediatric population. This is due to the inability of the pediatric population to perceive the symptoms and the lack of proper health care facilities to diagnose arrhythmias.

Radiofrequency ablation had now become the first-line treatment for many pediatric arrhythmic disorders associated with various congenital heart diseases due to its high success rate and low rate of complications. The small size of the heart is the most common challenging aspect that we come across in the pediatric population. Hence, it must be considered only for drug-refractory arrhythmias after failed medical management or heart failure due to tachycardiomyopathy. The varied anatomy associated with congenital heart diseases makes it tougher for catheter ablation and demands more technical expertise. The various venous anatomy abnormalities also pose a tougher challenge for electrophysiologists. The next most common challenge being, obtaining vascular access among the pediatric population. In many centers, there are few modifications done to the procedures while performing the pediatric procedures as compared to the adult population, which include the usage of fewer catheters than in the adult population during mapping and the usage of a transesophageal catheter to pace the left atrium and the usage of four French ablation catheters to avoid vascular complication. The most commonly encountered complications during pediatric radiofrequency ablation include complete heart block, venous access site issues, and cardiac perforation, with death being a rare complication, and is more frequent with small patient size and left-sided procedures.

In our study, we have demonstrated the safety and mid-term outcomes of arrhythmia ablation in the pediatric population aged two to 18 years. The important findings of our study are:

1. The acute procedural success rate was 100% irrespective of the underlying arrhythmia among the 36 patients who underwent ablation. Patients remained non-inducible at the end of the procedure. Kugler et al. [11] reported an acute success rate of 95% while Chiu et al. showed 92.6% [12].

2. AVNRT (44%) was noted more frequently than AVRT (39%) in our study. This is in contrast to other studies [13-14], where the most common arrhythmia in the pediatric population is AVRT (90%). The possible explanations for the lower prevalence of AVRT are: (a) absence of congenital heart disease in our study population, (b) average age of the study population was 12.86 ± 3.95 years, (c) less prevalence of pre-excitation syndrome in the general population.

3. The average weight of the study population was 36.3 ± 11.02 kg. The dosage of anesthetic medication was adjusted accordingly.

4. The most common AVRT was the left lateral pathway, mediated tachycardia. This is in concordance with a study by Wellens et al. [15], where it was found that 50% of accessory pathways were located on the left free wall. All left-sided accessory pathways were ablated by performing septal puncture (n=10, 28%). Septal puncture is safe even in younger patients, as two of our patients were aged less than seven years.

5. AVNRT is the most common arrhythmia in our series (n=16, 44%). The youngest patient was a two-year-old child with drug-refractory arrhythmia. None of our patients developed an AV node injury. Danford et al. [16] suggested the following steps to avoid injuring the AV node while ablating AVNRT in children: (a) temperature titration (50⁰ - 55⁰C), (b) target the lower third of Koch’s triangle, and (c) ablate as far away as possible from his bundle signals.

6. The mean number of RF pulses required for successful ablation was 2.3 ± 1.3 (excluding the atrial flutter case where an isthmus line was drawn).

7. Six patients underwent 3D EnSite velocity-guided ablation for atrial tachycardia, atrial flutter, and ventricular tachycardia. 3D-guided ablations are safe and effective in reducing radiation exposure both to the operator and to the patient.

8. Tachycardiomyopathy is seen even in pediatric arrhythmias. Two of our patients had LV dysfunction due to incessant atrial tachycardia and atrial flutter with a fast ventricular rate. In both cases, the ejection fraction recovered back to normal on follow-up.

9. The long-term success rate was 97.2%. One patient had an asymptomatic recurrence of the right posteroseptal pathway (2.7%). Lee et al. showed a recurrence rate of 4.7% during a long-term follow-up period of 86 ± 38 months [17] while Seixo et al. showed 12.9% over four years [18]. No mortality occurred in our study. Schaffer et al. reported an incidence of 0.12% deaths in their study on ablation with structurally normal hearts [19].

There are no long-term multicentre studies examining patients in adulthood after pediatric ablation; thus, concerns remain regarding scarring, late AV block development, latent coronary injury, and a small risk of malignancy secondary to radiation exposure.

Limitation of the study

Our study is a single-center, retrospective observational study. Hence, our experience cannot be extrapolated to the experience in the other parts of the world. Cryoablation, which is the preferred modality of ablation in children, is not currently available in our country. Hence, we are still using radiofrequency ablation for infants and children. Patients with complex congenital heart disease were not included in the study. This may be the reason for the less occurrence of AVRT, higher procedural success rate, and less recurrence. Long-term multicenter follow-up studies are essential to find out the late effects of RF ablation on the myocardium and radiation to patients.

## Conclusions

Arrhythmia in the pediatric population is one of the reasons for admission to the emergency department. Early recognition, effective management, and preventing the future recurrence of arrhythmias remain the cornerstone of the treatment protocol. The management of arrhythmia in the pediatric population ranges from anti-arrhythmic medications to invasive catheter ablation procedures. While most of the pediatric arrhythmias respond well to anti-arrhythmic medications, some of them are refractory to medical management. The long-term management of treatment-refractory pediatric arrhythmias is well-achieved by catheter ablation techniques. Despite its technical and anatomical challenges, catheter ablation in the pediatric population is a safe procedure to manage treatment-refractory arrhythmias and can be done with more feasibility and fewer complications when done in experienced hands.
